# Effects of in *IL-1B/IL-1RN* variants on the susceptibility to head and neck cancer in a chinese Han population

**DOI:** 10.1186/s12935-021-01750-0

**Published:** 2021-01-20

**Authors:** Yanhai Yin, Fen Li, Liangqian Tong, Chunru Chen, Bo Yuan

**Affiliations:** 1 Department of Nuclear medicine, Hainan Affiliated Hospital of Hainan Medical University, Hainan general Hospital, Haikou, Hainan Province China; 2 Department of Nuclear medicine, Haikou general Hospital, Haikou, Hainan Province China; 3 Department of General surgery, Hainan Affiliated Hospital of Hainan Medical University, Hainan general Hospital, #19 Xiuhua Road, Xiuying District, Haikou, Hainan Province 570311 China

**Keywords:** Head and neck cancer, *IL-1B* and *IL-1RN*, Genetic polymorphism, Susceptibility

## Abstract

**Background:**

The study aimed to evaluate the relationship of *IL-1B/IL-1RN* polymorphisms to the predisposition of head and neck cancer (HNC) in a Chinese Han population.

**Methods:**

Nine single-nucleotide polymorphisms (SNPs) in *IL-1B/IL-1RN* were genotyped based on Agena MassARRAY platform. Logistic regression models were used to analyze the genetic association between these SNPs and HNC risk by calculating odds ratios (ORs) and 95% confidence intervals (CI). Haplotype analysis were performed using Haploview program and logistic regression model.

**Results:**

The genetic association between rs1143643 in *IL-1B* and the higher risk of HNC was found (OR = 1.23, 95% CI 1.04–1.46) in the overall. *IL-1RN* rs17042888 was related to a reduced risk of HNC in the subjects aged > 46 years (OR = 0.70, 95% CI: 0.50–0.98) and in females (OR = 0.71, 95% CI 0.52–0.98), while rs1143643 increased the predisposition of HNC among females (OR = 1.76, 95% CI 1.13–2.74). Furthermore, rs1143643 had an increased susceptibility to thyroid carcinoma (OR = 1.61, 95% CI 1.10–2.34). Moreover, compared with stage I–II, the frequency of *IL-1RN* rs452204-AG genotype was lower in patients with stage III–IV.

**Conclusions:**

*IL-1B* (rs1143643) and *IL-1RN* (rs17042888 and rs452204) polymorphisms might be related to the individual susceptibility of HNC in the Chinese Han population. These results might help to improve the understanding of *IL-1B* and *IL-1RN* genes in the occurrence of HNC.

**Supplementary Information:**

The online version contains supplementary material available at 10.1186/s12935-021-01750-0.

## Introduction

Head and neck cancer (HNC) is the sixth commonly diagnosed cancer worldwide, including cancers of oral cavity, nasopharynx, larynx, and salivary glands [1]. According to the Global Cancer Statistics 2018, there were an estimated 873,734 new cases with thyroid, laryngeal and nasopharyngeal carcinoma and 208,829 deaths from these diseases [2]. In China, HNC causes more than 2.251 million cases and 77.5 thousands deaths annually [3]. The pathogenesis of HNC has not been fully elucidated. It is known that environmental exposures to smoking and alcohol consumption are identified as the major risk factors for HNC [4, 5]. However, not all individuals with these risk factors will eventually develop HNC, which indicates that genetic factors may play a critical role in the carcinogenesis and progression of HNC. Cumulative evidence indicates that genetic variants contribute to the risk of HNC [6–8]. However, a large number of risk polymorphisms associated with HNC predisposition have not been discovered.

Interleukin-1 (IL-1) is an inflammatory cytokine that plays a key role in the incidence and development of tumor [9]. IL-1 family includes mainly of IL-1α, IL‐1β (pro‐inflammatory effect) and IL‐1 receptor antagonist (IL‐1RN, anti‐inflammatory effect). The tumor progression driven by IL-1 is related to the promotion of angiogenesis[10]. IL-β participates in the suppression of adaptive antitumor immune responses by regulating myeloid-derived suppressor cells [11]. Furthermore, IL-1β promotes epithelial to mesenchymal transition to support metastasis [12]. IL‐1RN, as a natural IL-1 inhibitor, serves as a decoy target by binding to its receptor without activating signal conversion. IL-1RN can target the tumor microenvironment by interfering in IL-1 biology[13]. Several studies have reported the relationship between genetic polymorphisms of IL-1 family genes and the risk of various cancers, including breast, gastric, colorectal, and cervical cancer [14–16], but HNC has not reported.

Here, five SNPs in *IL-1B* and four SNPs in *IL-1RN* were genotyped to discover the genetic association between *IL-1B/ IL-1RN* polymorphisms and the risk of HNC occurrence among the Chinese Han population. The heterogeneity of associations between subgroups defined by age (> 46 years and ≤ 46 years) and sex (males and females) were assessed. Furthermore, we explored the relationship of *IL-1B/IL-1RN* polymorphisms with clinical features of HNC, including HNC type, stage, and lymph nodes metastasis.

## Materials and methods

### Study population

We recruited 535 HNC patients and 538 healthy controls from Shaanxi Provincial Cancer Hospital. All participants were genetically unrelated ethnic Han Chinese population. HNC patients were diagnosed and histopathologically confirmed by two pathologists. These cases who had previous history of other cancers, chronic diseases involving vital organs, inflammation, infection, or other autoimmune diseases were excluded. HNC patients had not received chemotherapy or radiation before sample collection. Healthy controls were enrolled from the physical examination center at the same hospital. The inclusion criterion for healthy controls was no cancer history, no acute or chronic disease, and no inflammatory or immune diseases. Demographic and clinical data were obtained from the questionnaires and medical records. The study was approved by the Ethics Committee of Hainan People’s Hospital and complied with the Declaration of Helsinki. All subjects gave informed consent.

### DNA extraction and SNPs genotyping

Approximately 5 mL samples were gathered in EDTA vacutainer tubes from all subjects. Genomic DNA was extracted with GoldMag DNA Extraction Kits (GoldMag Co. Ltd, Xi′an, China) and stored at − 20 ℃ before genotyping. Based on the NCBI dbSNP database and 1000 Genomes Project database, candidate SNPs with the minor allele frequency (MAF) > 0.05 low linkage disequilibrium (r^2^ < 0.8), and the call rate > 95% were selected. Finally, nine SNPs in the *IL-1B* (rs1143643, rs1143630, rs1143627, rs16944 and rs1143623) and *IL-1RN* (rs17042888, rs315919, rs3181052 and rs452204) genes were included. The genotypes of *IL-1B* and *IL-1RN* polymorphisms were detected using Agena MassARRAY platform (Agena, San Diego, CA, USA). Primers design and data management were conducted by supporting software. The primers were presented in Additional file 1: Table S1. Approximately10% of the study subjects were repeated genotyping for the quality control, and the results were consistent.

### Statistical analysis

The characteristics of subjects were displayed as mean ± standard deviation (SD) or frequency (percentage). The age and gender distribution between HNC patients and healthy controls were evaluated by χ^2^ test or sample t test, respectively. The goodness of fit χ^2^ was used to test the Hardy–Weinberg equilibrium (HWE) of SNPs among controls. Logistic regression analysis adjusted for age and gender was applied to assess the genetic association between *IL-1B/IL-1RN* variants and HNC risk by calculating odds ratios (ORs) and 95% confidence intervals (CI). Linkage disequilibrium (LD) and haplotype analysis were evaluated using Haploview program and logistic regression model. SPSS 20.0 (SPSS Inc., Chicago, IL, USA), PLINK 1.0.7, Haploview 4.2 software were used for data analyses. Two-tailed *p* < 0.05 was considered statistically significant.

## Results

### Study population

The characteristics of HNC patients and healthy controls were shown in Table 1. A total of 535 patients (46.8 ± 15.1 years) and 538 controls (46.9 ± 13.8 years) were included. The ratio of male to female was 2:3 in the cases and controls. There was no significant difference in age and gender distribution between the two groups (*p* = 0.875 and 0.908, respectively). Among the 535 patients, 75 (14.0%) were nasopharyngeal carcinoma, 398 (74.4%) were thyroid cancer, 43 (8.0%) were laryngeal carcinoma, and 19 (3.6%) were parotid gland cancer.

**Table 1:** Characteristics of patients with head and neck cancer and health controls

Variables	Cases	Control	*P*
N	535	538	
Age			
Mean ± SD, years	46.8 ± 15.1	46.9 ± 13.8	0.875
> 46 years	296 (55.3%)	301 (55.9%)	
≤ 46 years	239 (44.7%)	237 (44.1%)	
Gender			0.908
Male	205 (38.3%)	208 (38.7%)	
Female	330 (61.7%)	330 (61.3%)	
Lymph nodes metastasis			
Yes	101 (18.9%)		
No	82 (15.3%)		
Unavailable	352 (65.8%)		
Stage			
I-II	140 (26.2%)		
III-IV	34 (6.4%)		
Unavailable	361 (67.5%)		
Type of head and neck cancer			
Nasopharyngeal carcinoma	75 (14.0%)		
Thyroid cancer	398 (74.4%)		
Laryngeal cancer	43 (8.0%)		
Parotid gland carcinoma	19 (3.6%)		

*p* values were calculated by χ^2^ test or the Student’s t test

*p < 0.05 indicates statistical significance

### The contribution of IL-1B/IL-1RN polymorphisms to the risk of HNC

The detailed information of these SNPs in *IL-1B/IL-1RN* including position, MAF, the call rate of genotyping and HWE were presented in Table 2. The MAF of these SNPs were all > 5% and the genotyping rate exceeded 99.5%. Except for rs1143623 (*p* < 0.001), the genotype frequencies of other variants were in HWE among the control group. The prevalence of *IL-1B* rs1143643-T allele in HNC patients (52.8%) was higher than that in controls (47.6%). Furthermore, the genetic association was found between rs1143643 and the increased risk of HNC (OR = 1.23, 95% CI 1.04–1.46, *p* = 0.016).

**Table 2:** The details of candidate SNPs in *IL-1B/IL-1RN* and the allele model for the association between these polymorphisms and the risk of head and neck cancer

Gene	SNP ID	Chr: Position	Alleles (Ref/Alt)	MAF		Call rate	HWE			OR (95% CI)	*p* ^b^
				Cases	Controls		O(HET)	E(HET)	*p* ^a^		
*IL-1B*	rs1143643	2:112,830,725	C/T	0.528	0.476	100.0%	0.506	0.499	0.796	1.23 (1.04–1.46)	0.016*
*IL-1B*	rs1143630	2:112,834,078	G/T	0.163	0.161	100.0%	0.273	0.270	0.874	1.01 (0.81–1.28)	0.908
*IL-1B*	rs1143627	2:112,836,810	G/A	0.520	0.493	99.8%	0.496	0.500	0.863	1.11 (0.94–1.32)	0.210
*IL-1B*	rs16944	2:112,837,290	A/G	0.525	0.494	99.9%	0.494	0.500	0.796	1.13 (0.96–1.34)	0.154
*IL-1B*	rs1143623	2:112,838,252	C/G	0.409	0.417	100.0%	0.403	0.486	< 0.001*		
*IL-1RN*	rs17042888	2:113,104,596	G/A	0.242	0.271	100.0%	0.413	0.396	0.383	0.86 (0.71–1.04)	0.120
*IL-1RN*	rs315919	2:113,118,636	T/G	0.375	0.409	100.0%	0.480	0.483	0.859	0.87 (0.73–1.03)	0.105
*IL-1RN*	rs3181052	2:113,128,472	A/G	0.392	0.421	100.0%	0.470	0.488	0.426	0.89 (0.75–1.05)	0.165
*IL-1RN*	rs452204	2:113,131,484	A/G	0.326	0.354	99.5%	0.448	0.458	0.637	0.88 (0.74–1.05)	0.167

*SNP* Single nucleotide polymorphism, * MAF* Minor allele frequency,* HWE* Hardy-Weinberg equilibrium, * O(HET)* Observed heterozygosity frequency, *E(HET)* Expected heterozygosity frequency

*p* ^a^ values were calculated by χ^2^ test

*p* ^b^ values were calculated by logistic regression analysis with adjustments for age and gender

*p < 0.05 indicates statistical significance

The genotype frequency distribution of these selected SNPs in patients and controls was shown in Table 3. The frequency difference of rs1143643-TT genotype was observed between HNC patients (26.9%) and healthy controls (22.3%). Carriers with TT genotype had an increased susceptibility to HNC than CC genotype (OR = 1.54, 95% CI 1.09–2.17, *p* = 0.015). Genetic model analysis revealed that rs1143643 in *IL-1B* was related to the higher risk of HNC under the dominant (OR = 1.38, 95% CI 1.04–1.82, *p* = 0.026) and additive (OR = 1.24, 95% CI 1.04–1.47, *p* = 0.015) models. No significant association of other SNPs in *IL-1B/IL-1RN* with HNC risk was observed.

**Table 3:** The effect of genetic variants in *IL-1B/IL-1RN* on the susceptibility to head and neck cancer

Gene	SNP ID	Model	Genotype	Case	Control	Adjusted by age and gender	
						OR (95% CI)	*p*
*IL-1B*	rs1143643	Genotype	CC	114	146	1	
			CT	277	272	1.30 (0.97–1.75)	0.080
			TT	144	120	1.54 (1.09–2.17)	0.015*
		Dominant	CC	114	146	1	
			CT-TT	421	392	1.38 (1.04–1.82)	0.026*
		Recessive	CC-CT	391	418	1	
			TT	144	120	1.28 (0.97–1.70)	0.080
		Log-additive				1.24 (1.04–1.47)	0.015*
*IL-1B*	rs1143630	Genotype	GG	378	378	1	
			GT	140	147	0.95 (0.73–1.25)	0.736
			TT	17	13	1.31 (0.63–2.74)	0.473
		Dominant	GG	378	378	1	
			GT-TT	157	160	0.98 (0.76–1.28)	0.898
		Recessive	GG-GT	518	525	1	
			TT	17	13	1.33 (0.64–2.76)	0.449
		Log-additive				1.02 (0.81–1.28)	0.898
*IL-1B*	rs1143627	Genotype	GG	118	139	1	
			GA	278	266	1.23 (0.91–1.66)	0.171
			AA	139	131	1.25 (0.89–1.76)	0.201
		Dominant	GG	118	139	1	
			GA-AA	417	397	1.24 (0.93–1.64)	0.138
		Recessive	GG-GA	396	405	1	
			AA	139	131	1.09 (0.82–1.43)	0.559
		Log-additive				1.12 (0.94–1.33)	0.205
*IL-1B*	rs16944	Genotype	AA	116	139	1	
			AG	276	265	1.25 (0.93–1.68)	0.146
			GG	143	133	1.29 (0.92–1.81)	0.146
		Dominant	AA	116	139	1	
			AG-GG	419	398	1.26 (0.95–1.67)	0.107
		Recessive	AA-AG	392	404	1	
			GG	143	133	1.11 (0.84–1.46)	0.462
		Log-additive				1.13 (0.96–1.34)	0.151
*IL-1RN*	rs17042888	Genotype	GG	310	281	1	
			GA	191	222	0.78 (0.61–1.00)	0.053
			AA	34	35	0.88 (0.54–1.45)	0.622
		Dominant	GG	310	281	1	
			GA-AA	255	257	0.79 (0.62–1.01)	0.060
		Recessive	GG-GA	501	503	1	
			AA	34	35	0.98 (0.60–1.59)	0.925
		Log-additive				0.86 (0.70–1.04)	0.119
*IL-1RN*	rs315919	Genotype	TT	204	189	1	
			TG	261	258	0.94 (0.72–1.22)	0.627
			GG	70	91	0.71 (0.49–1.03)	0.072
		Dominant	TT	204	189	1	
			TG-GG	331	349	0.88 (0.69–1.13)	0.307
		Recessive	TT-TG	465	447	1	
			GG	70	91	0.74 (0.53–1.04)	0.080
		Log-additive				0.86 (0.73–1.03)	0.103
*IL-1RN*	rs3181052	Genotype	AA	196	185	1	
			AG	259	253	0.97 (0.74–1.26)	0.799
			GG	80	100	0.76 (0.53–1.08)	0.123
		Dominant	AA	196	185	1	
			AG-GG	339	353	0.91 (0.71–1.16)	0.441
		Recessive	AA-AG	455	438	1	
			GG	80	100	0.77 (0.56–1.06)	0.113
		Log-additive				0.89 (0.75–1.05)	0.169
*IL-1RN*	rs452204	Genotype	AA	237	226	1	
			AG	243	240	0.97 (0.75–1.25)	0.793
			GG	52	70	0.71 (0.47–1.06)	0.093
		Dominant	AA	237	226	1	
			AG-GG	295	310	0.91 (0.71–1.16)	0.433
		Recessive	AA-AG	480	466	1	
			GG	52	70	0.72 (0.49–1.06)	0.092
		Log-additive				0.88 (0.74–1.05)	0.166

*SNP* single nucleotide polymorphism,* OR* odds ratio,* 95% CI* 95% confidence interval

*p* values were calculated by logistic regression analysis with adjustments for age and gender

*p < 0.05 respects the data is statistically significant

### Stratified analysis by age and gender for the relationship of IL-1B/IL-1RN variants with HNC risk

Stratified analysis was used to assess the heterogeneity of relationship between subgroups defined by age (> 46 years and ≤ 46 years) and sex (males and females), and the results were presented in Table 4. In age stratification, we found that *IL-1RN* rs17042888 reduced the risk of HNC (GA vs. GG, OR = 0.70, 95% CI 0.50–0.98, *p* = 0.040) in subjects aged > 46 years. In females, the relationship between rs1143643 in *IL-1B* and higher predisposition of HNC was found in the allele (OR = 1.31, 95% CI 1.05–1.62, *p* = 0.015), genotype (OR = 1.76, 95% CI 1.13–2.74, *p* = 0.013), dominant (OR = 1.54, 95% CI 1.07–2.21, *p* = 0.021) and additive (OR = 1.32, 95% CI 1.06–1.65, *p* = 0.014) models. Whereas, *IL-1RN* rs17042888 was related to the lower risk of HNC among females (GA vs. GG, OR = 0.71, 95% CI 0.52–0.98, *p* = 0.038; and GA-AA vs. GG, OR = 0.73, 95% CI 0.53–0.99, *p* = 0.042).

**Table 4:** Stratification by age and gender for the effect of genetic variants in *IL-1B/IL-1RN* on the susceptibility of head and neck cancer

Gene	SNP ID	Model	Genotype	Case	Control	OR (95% CI)	*p*	Case	Control	OR (95% CI)	*p*
Age stratification				> 46 years				≤ 46 years			
*IL-1RN*	rs17042888	Allele	G	449	439	1		362	345	1	
			A	143	163	0.86 (0.66–1.11)	0.248	116	129	0.86 (0.64–1.15)	0.298
		Genotype	GG	176	157	1		134	124	1	
			GA	97	125	0.70 (0.50–0.98)	0.040*	94	97	0.91 (0.63–1.33)	0.627
			AA	23	19	1.07 (0.56–2.05)	0.831	11	16	0.64 (0.29–1.44)	0.285
		Dominant	GG	176	157	1		134	124	1	
			GA-AA	120	144	0.75 (0.54–1.04)	0.080	105	113	0.87 (0.61–1.25)	0.463
		Recessive	GG-GA	273	282	1		228	221	1	
			AA	23	19	1.24 (0.66–2.32)	0.510	11	16	0.67 (0.30–1.48)	0.321
		Log-additive				0.86 (0.67–1.12)	0.262			0.86 (0.64–1.16)	0.322
Gender stratification				Males				Females			
*IL-1B*	rs1143643	Allele	C	203	219	1		302	346	1	
			T	207	198	1.12 (0.85–1.48)	0.406	358	314	1.31 (1.05–1.62)	0.015*
		Genotype	CC	50	57	1		64	89	1	
			CT	103	107	1.13 (0.71–1.81)	0.600	174	168	1.44 (0.98–2.12)	0.063
			TT	52	47	1.26 (0.73–2.19)	0.401	92	73	1.76 (1.13–2.74)	0.013*
		Dominant	CC	50	57	1		64	89	1	
			CT-TT	155	151	1.17 (0.76–1.83)	0.476	266	241	1.54 (1.07–2.21)	0.021*
		Recessive	CC-CT	153	161	1		238	257	1	
			TT	52	47	1.16 (0.74–1.83)	0.510	92	73	1.36 (0.96–1.94)	0.086
		Log-additive				1.13 (0.86–1.48)	0.401			1.32 (1.06–1.65)	0.014*
*IL-1RN*	rs17042888	Allele	G	306	306	1		505	478	1	
			A	104	110	0.95 (0.69–1.29)	0.724	155	182	0.81 (0.63–1.03)	0.088
		Genotype	GG	115	112	1		195	169	1	
			GA	76	82	0.90 (0.60–1.36)	0.624	115	140	0.71 (0.52–0.98)	0.038*
			AA	14	14	0.97 (0.44–2.13)	0.939	20	21	0.83 (0.43–1.58)	0.571
		Dominant	GG	115	112	1		195	169	1	
			GA-AA	90	96	0.91 (0.62–1.35)	0.646	135	161	0.73 (0.53–0.99)	0.042*
		Recessive	GG-GA	191	194	1		310	309	1	
			AA	14	14	1.01 (0.47–2.18)	0.977	20	21	0.95 (0.51–1.80)	0.884
		Log-additive				0.95 (0.69–1.29)	0.721			0.80 (0.63–1.03)	0.087

*SNP* single nucleotide polymorphism, *OR* odds ratio, *95% CI* 95% confidence interval

*p* values were calculated by logistic regression analysis with adjustments for age and gender

*p < 0.05 respects the data is statistically significant

### The relationship of IL-1B/IL-1RN variants with clinical features of HNC

Furthermore, we investigated the relationship between *IL-1B/IL-1RN* SNPs and the clinical features of HNC, including HNC type, stage, and lymph nodes metastasis. The effect of *IL-1B/IL-1RN* variants on the susceptibility to nasopharyngeal carcinoma or thyroid carcinoma was assessed, and the results were displayed in Table 5. Rs1143643 in *IL-1B* increased the susceptibility to thyroid carcinoma under the allele (OR = 1.26, 95% CI 1.05–1.52, *p* = 0.013), genotype (OR = 1.61, 95% CI 1.10–2.34, *p* = 0.014), dominant (OR = 1.42, 95% CI 1.04–1.94, *p* = 0.027), and additive (OR = 1.27, 95% CI 1.05–1.53, *p* = 0.015) models. Moreover, the relationship between *IL-1B/IL-1RN* variants and clinical stages in HNC patients was evaluated. The results displayed that compared with stage I–II, patients with stage III–IV had a lower frequency of *IL-1RN* rs452204-AG genotype (18.2% vs. 48.6%, OR = 0.28, 95% CI 0.10–0.78, *p* = 0.015; Table 6). There was no significant relationship of *IL-1B/IL-1RN* SNPs with lymph nodes metastasis (data not shown).

**Table 5:** The effect of genetic variants in *IL-1B/IL-1RN* on the susceptibility of nasopharyngeal carcinoma and thyroid carcinoma

Gene	SNP ID	Model	Genotype	Control	Nasopharyngeal carcinoma			Thyroid carcinoma		
					Case	OR (95% CI)	*p*	Case	OR (95% CI)	*p*
*IL-1B*	rs1143643	Allele	C	564	87	1		371	1	
			T	512	72	1.02 (0.72–1.43)	0.924	425	1.26 (1.05–1.52)	0.013*
		Genotype	CC	146	20	1		82	1	
			CT	272	38	1.03 (0.51–2.07)	0.946	207	1.61 (1.1–2.34)	0.014*
			TT	120	17	1.06 (0.59–1.91)	0.841	109	1.34 (0.97–1.86)	0.080
		Dominant	CC	146	20	1		82	1	
			CT-TT	392	55	1.05 (0.60–1.83)	0.863	316	1.42 (1.04–1.94)	0.027*
		Recessive	CC-CT	418	58	1		289	1	
			TT	120	17	0.99 (0.55–1.77)	0.961	109	1.31 (0.97–1.78)	0.078
		Log-additive				1.01 (0.72–1.44)	0.936		1.27 (1.05–1.53)	0.015*

*SNP* single nucleotide polymorphism, *OR* odds ratio,* 95% CI* 95% confidence interval

*p* values were calculated by logistic regression analysis with adjustments for age and gender

*p < 0.05 respects the data is statistically significant

**Table 6:** Relationship of clinical stage with *IL-1B/IL-1RN* variants in head and neck cancer patients

Gene	SNP ID	Model	Genotype	Head and neck cancer			
				III-IV	I-II	OR (95% CI)	*p*
*IL-1RN*	rs452204	Allele	A	48	190	1	
			G	18	90	0.79 (0.44–1.44)	0.442
		Genotype	AA	21	61	1	
			AG	6	68	0.28 (0.10–0.78)	0.015*
			GG	6	11	1.71 (0.53–5.47)	0.369
		Dominant	AA	21	61	1	
			AG-GG	12	79	0.49 (0.22–1.13)	0.094
		Recessive	AA-AG	27	129	1	
			GG	6	11	2.70 (0.87–8.37)	0.085
		Log-additive				0.87 (0.47–1.61)	0.662

*SNP* single nucleotide polymorphism, *OR* odds ratio,* 95% CI* 95% confidence interval

*p* values were calculated by logistic regression analysis with adjustments for age and gender

*p < 0.05 respects the data is statistically significant

### The association of IL-1B haplotype with HNC risk

**Fig. 1:**
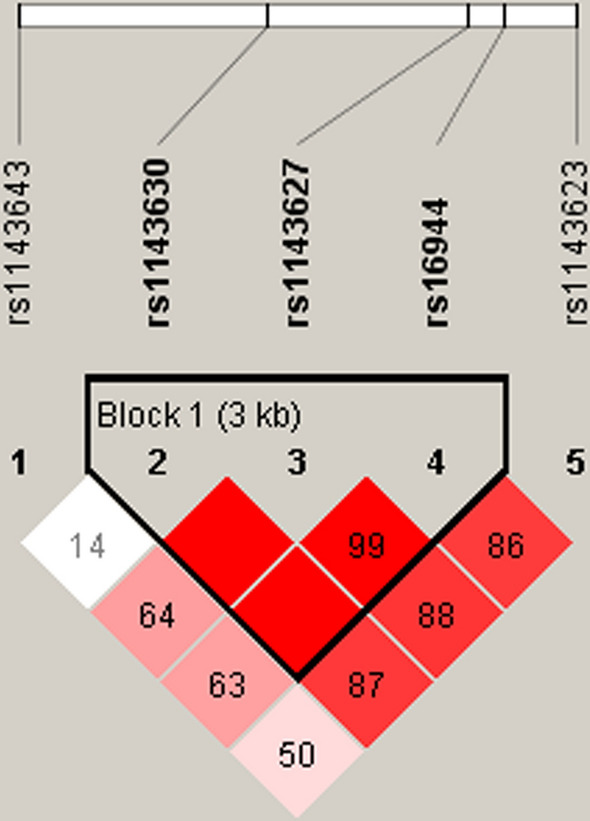
Linkage disequilibrium (LD) analysis of IL-1B SNPs measured by D¢. The block structure was assessed using Haploview 4.2 software

Additionally, haplotype analysis was performed to investigate whether *IL-1B/IL-1RN* variants were in linkage disequilibrium. The result revealed that there were one block and three haplotypes (GAG, TGA and GGA) in the three *IL-1B* SNPs (rs1143630, rs1143627 and rs16944), as shown in Fig. 1. The haplotype frequencies of *IL-1B* haplotypes in the in the case group and the control group were shown in Additional file 1: Table S2. The contribution of *IL-1B* haplotypes to HNC susceptibility was analyzed; however, there was no significant correlation between the haplotypes of *IL-1B* and HNC risk (*p* > 0.05) (Additional file 2).

## Discussion

In this study, the genetic association of eight SNPs in *IL-1B/ IL-1RN* with HNC susceptibility was evaluated, which displayed that *IL-1B* rs1143643 increased the risk of HNC in the overall. *IL-1RN* rs17042888 was associated with a reduced risk of HNC in subjects aged > 46 years and in females. The relationship between rs1143643 and higher predisposition of HNC was found among females. Furthermore, rs1143643 was associated with increased susceptibility of thyroid carcinoma, not nasopharyngeal carcinoma. Moreover, compared with stage I–II, patients with stage III–IV had a lower frequency of *IL-1RN* rs452204-AG genotype. This is the first study to assess the effect of *IL-1B/ IL-1RN* SNPs on HNC predisposition.

IL-1 plays a role in the genesis and progression of tumors, including promoting tumor angiogenesis, driving non-resolving inflammatory microenvironment, inducing myeloid-derived suppressor cells, activating IL-1-IL-17 pathway, and maintaining the activity of immunosuppressive tumor-associated macrophage [9]. Pervious study reported the relation between IL1-RN and the degree of HNC differentiation [17]. Overexpression of IL-1 activity increases the growth and metastasis of HNC [18]. IL1 was identified to be associated with the increased anti-tumor efficacy of cetuximab in HNC [19]. The intervention of IL-1β-CCL22-CCR4 signaling axis might provide a new treatment strategy for HNC [20]. These studies supported that IL-1 had a crucial role in the occurrence and development of HNC. Here, we investigated the contribution of *IL-1B/ IL-1RN* variants to HNC risk among Chinese Han population for the first time.

*IL1B* rs1143643 C > T, located in the intron region, might have a functional effect on the selected eQTL hits, enhancer histone marks, motifs changed, and DNAse, as predicted by HaploReg v4.1. Previously, *IL1B* rs1143643 was related to various diseases, such as neonatal sepsis, pediatric asthma, and cervical cancer [21–23]. Here, we found that the prevalence of *IL-1B* rs1143643-T allele in HNC patients (52.8%) was higher than that in the controls (47.6%). *IL-1B* rs1143643 increased the risk of HNC under the multiple models. Considering that age and gender are risk factors for HNC [24], we conducted a further stratified analysis to assess the heterogeneity of the association between subgroups defined by age and sex. We found that *IL-1RN* rs17042888 might be a protective factor for HNC in subjects aged > 46 years and in females. Similarly, a study revealed that *IL-1RN* rs17042888 could reduce the risk of thyroid carcinoma [25]. Moreover, the relationship between rs1143643 and higher predisposition of HNC was found among females. These results indicated that the genetic association between *IL-1B/ IL-1RN* SNPs and HNC predisposition might be related to age and gender.

Furthermore, the correlation between *IL-1B/IL-1RN* variants and the clinical features of HNC, including HNC type, stage, and lymph nodes metastasis was also evaluated. IL-1β exerts strong antitumor effects on thyroid carcinoma by inhibiting proliferation and invasiveness [26]. *IL1B* rs1143643 was related to lymph node metastasis of papillary thyroid carcinoma in Korean patients [27]. We found that rs1143643 was associated with increased susceptibility to thyroid carcinoma. Moreover, compared with stage I–II, patients with stage III–IV had a lower frequency of *IL-1RN* rs452204-AG genotype. However, we did not find a significant relationship between *IL-1B/ IL-1RN* variants and lymph node metastasis of HNC.

Nonetheless, some potential limitations cannot be ignored. First, the sample size of laryngeal carcinoma and parotid gland cancer was relatively small, therefore the correlation between *IL-1B/ IL-1RN* SNPs and the risk of laryngeal carcinoma and parotid gland cancer was not analyzed. Second, the subjects in this study were recruited from one hospital, so the inherent selection bias cannot be completely excluded. Here, we matched the age, gender, and frequency between cases and controls, and adjusted for potential confounding factors to minimize the bias. Third, due to the incomplete information, the interaction between environmental and genetic factors in the risk of HNC was not assessed in this study.

## Conclusions

In conclusion, our results indicated that *IL-1B* (rs1143643) and *IL-1RN* (rs17042888 and rs452204) polymorphisms might contribute to the individual predisposition to HNC in Chinese Han population. These results might help to enhance the understanding of *IL-1B* and *IL-1RN* genes in the occurrence and development of HNC. Further studies on various different ethnic groups and a large sample sizes are required to validate our findings.

## Supplementary information

**Additional file1: Table S1.** The detail of PCR primers and UEP sequence for genetic variants in IL-1B/IL-1RN. ** Table S2.** Haplotype frequencies of IL-1B gene and the correlation with the risk of head and neck cancer.

**Additional file 2:** The results of genotype.

## Data Availability

The datasets used during the current study are available from the corresponding author on a reasonable request.

## References

[1] Cohen N, Fedewa S, Chen AY (2018). Epidemiology and Demographics of the Head and Neck Cancer Population. Oral Maxillofacial Surgery Clinics North America.

[2] Bray F, Ferlay J, Soerjomataram I (2018). Global cancer statistics 2018: GLOBOCAN estimates of incidence and mortality worldwide for 36 cancers in 185 countries. Cancer J Clin.

[3] Chen W, Zheng R, Baade PD (2016). Cancer statistics in China, 2015. Cancer J Clin.

[4] Zhou J, Michaud DS, Langevin SM (2013). Smokeless tobacco and risk of head and neck cancer: evidence from a case-control study in New England. Int J cancer.

[5] Kawakita D, Matsuo K (2017). Alcohol and head and neck cancer. Cancer Metastasis Rev.

[6] Li L, Zhang ZT (2019). Genetic Association between NFKBIA and NFKB1 Gene Polymorphisms and the Susceptibility to Head and Neck Cancer: A Meta-Analysis. Disease Markers.

[7] Dylawerska A, Barczak W, Wegner A (2017). Association of DNA repair genes polymorphisms and mutations with increased risk of head and neck cancer: a review. Med Oncol&nbsp;.

[8] Kiczmer P, Prawdzic Seńkowska A, Strzelczyk JK (2018). The role of MGMT polymorphisms rs12917 and rs11016879 in head and neck cancer risk and prognosis. Acta Biochim Pol.

[9] Mantovani A, Barajon I, Garlanda C (2018). IL-1 and IL-1 regulatory pathways in cancer progression and therapy. Immunological Rev.

[10] Voronov E, Carmi Y, Apte RN (2014). The role IL-1 in tumor-mediated angiogenesis. Front Physiol.

[11] Elkabets M, Ribeiro VS, Dinarello CA (2010). IL-1β regulates a novel myeloid-derived suppressor cell subset that impairs NK cell development and function. Eur J Immunol.

[12] Voronov E, Dotan S, Krelin Y (2013). Unique Versus Redundant Functions of IL-1α and IL-1β in the Tumor Microenvironment. Front&nbsp; Immunol.

[13] Voronov E, Apte RN (2017). Targeting the Tumor Microenvironment by Intervention in Interleukin-1 Biology. Curr Pharm Design.

[14] Wang J, Shi Y, Wang G (2019). The association between interleukin-1 polymorphisms and their protein expression in Chinese Han patients with breast cancer. Mol Genet Genom Med.

[15] Abbasian MH, Abbasi B, Ansarinejad N (2018). Association of interleukin-1 gene polymorphism with risk of gastric and colorectal cancers in an Iranian population. Iranian J Immunol.

[16] Zidi S, Sghaier I, Zouidi F (2015). Interleukin-1 Gene Cluster Polymorphisms and its Haplotypes may Predict the Risk to Develop Cervical Cancer in Tunisia. Pathology Oncol Res.

[17] Westin U, Nyström M, Ljungcrantz I (2002). The presence of elafin, SLPI, IL1-RA and STNFalpha RI in head and neck squamous cell carcinomas and their relation to the degree of tumour differentiation. Mediat Inflamm.

[18] von Biberstein SE, Spiro JD, Lindquist R (1996). Interleukin-1 receptor antagonist in head and neck squamous cell carcinoma. Archives&nbsp; Otolaryngol Head Neck Surg.

[19] Espinosa-Cotton M, Rodman Iii SN, Ross KA (2019). Interleukin-1 alpha increases anti-tumor efficacy of cetuximab in head and neck squamous cell carcinoma. J Immunother Cancer.

[20] Huang YH, Chang CY, Kuo YZ (2019). Cancer-associated fibroblast-derived interleukin-1β activates protumor C-C motif chemokine ligand 22 signaling in head and neck cancer. Cancer Sci.

[21] Mustarim M, Yanwirasti Y, Jamsari J (2019). Association of Gene Polymorphism of Bactericidal Permeability Increasing Protein Rs4358188, Cluster of Differentiation 14 Rs2569190, Interleukin 1β Rs1143643 and Matrix Metalloproteinase-16 Rs2664349 with Neonatal Sepsis. Open Access Macedonian J Med Sci.

[22] Sobkowiak P, Wojsyk-Banaszak I, Kowalewska M (2017). Interleukin 1β polymorphism and serum level are associated with pediatric asthma. Pediatric Pulmonol.

[23] Pontillo A, Bricher P, Leal VN (2016). Role of inflammasome genetics in susceptibility to HPV infection and cervical cancer development. Journal&nbsp;Med Virol.

[24] Rettig EM, D’Souza G (2015). Epidemiology of head and neck cancer. Surg Oncol Clin N Am.

[25] Li H, Wu Y, Zhao R, et al: IL-1RN gene polymorphisms reduces thyroid cancer risk in Chinese Han population. 2020. 10.1002/mc.23244 32790111

[26] Yip I, Pang XP, Berg L (1995). Antitumor actions of interferon-gamma and interleukin-1 beta on human papillary thyroid carcinoma cell lines. J Clin Endocrinol Metab.

[27] Ban JY, Kim MK, Park SW (2012). Interleukin-1 beta polymorphisms are associated with lymph node metastasis in Korean patients with papillary thyroid carcinoma. Immunol Investig.

