# Supplementary material for: Effects of in IL-1B/IL-1RN variants on the susceptibility to head and neck cancer in a chinese Han population
Source: Cancer Cell Int. 2021 Jan 20;21:59. doi: 10.1186/s12935-021-01750-0 (PMC7816368; doi:10.1186/s12935-021-01750-0)
Supplement: Supplementary file 1 — Additional file1: Table S1. The detail of PCR primers and UEP sequence for genetic variants in IL-1B/IL-1RN. Table S2. Haplotype frequencies of IL-1B gene and the correlation with the risk of head and neck cancer. [file 12935_2021_1750_MOESM1_ESM.docx]

Table S1. The detail of PCR primers and UEP sequence for genetic variants in *IL-1B/IL-1RN*

| Gene | SNPs | First Primer(5'-3') | Second Primer (5'-3') | UEP_DIR | UEP SEQ (5'-3') |
| --- | --- | --- | --- | --- | --- |
| *IL-1B* | rs1143643 | ACGTTGGATGACTCCTGAGTTGTAACTGGG | ACGTTGGATGCCTCAGCATTTGGCACTAAG | F | GGGCCCCCAACTTTC |
| *IL-1B* | rs1143630 | ACGTTGGATGAGATTATCCCTCTCTGAAGC | ACGTTGGATGTCTTGAGTCTGCCTCTAACC | F | AGCTCAAGGAGGTTAAG |
| *IL-1B* | rs1143627 | ACGTTGGATGTTGTGCCTCGAAGAGGTTTG | ACGTTGGATGTCTCAGCCTCCTACTTCTGC | F | gtTCCCTCGCTGTTTTTAT |
| *IL-1B* | rs16944 | ACGTTGGATGAGAGGCTCCTGCAATTGACA | ACGTTGGATGCTGTCTGTATTGAGGGTGTG | R | AATTGACAGAGAGCTCC |
| *IL-1B* | rs1143623 | ACGTTGGATGATGTGCCAGGTATCGTGCTC | ACGTTGGATGACCTATTTCCCTCGTGTCTC | F | tttaGTGCTCGCTCTGCATTAT |
| *IL-1RN* | rs17042888 | ACGTTGGATGCTACTTGCTCAGCACCATAC | ACGTTGGATGTGGAGTTGGAGTCTTGTTGG | R | agcGGTGTTGAAATCCCAAAA |
| *IL-1RN* | rs315919 | ACGTTGGATGCACACAAATCCTAACCGGAG | ACGTTGGATGCCAGACAATAAAGCAAGCAG | R | tTTGCAAACTGGCAGCTTATA |
| *IL-1RN* | rs3181052 | ACGTTGGATGACAGTCCCCATATCTGGAAG | ACGTTGGATGCTTTATGTTTGTCTGGGCCG | R | cttaACTCATACACCCACAGAGCC |
| *IL-1RN* | rs452204 | ACGTTGGATGTAGACTTAGCCACGTGACTG | ACGTTGGATGAAAAGAGCCTCAACATGCAG | R | gcccATAGGATGATGCAAGCAGAAGT |

SNP, Single nucleotide polymorphism; UEP, Unextended mini sequencing primer; DIR, direction; SEQ, sequence.

Table S2. Haplotype frequencies of *IL-1B* gene and the correlation with the risk of head and neck cancer

| SNP | Haplotype | Frequency | | χ^2^ | *p^a^* | Adjusted by age and gender | |
| --- | --- | --- | --- | --- | --- | --- | --- |
|  |  | Case | Control |  |  | OR (95% CI) | *p^b^* |
| rs1143630\|rs1143627\|rs16944 | GAG | 0.520 | 0.492 | 1.682 | 0.195 | 1.12 (0.94–1.33) | 0.190 |
| rs1143630\|rs1143627\|rs16944 | TGA | 0.837 | 0.839 | 0.013 | 0.908 | 0.99 (0.78–1.24) | 0.898 |
| rs1143630\|rs1143627\|rs16944 | GGA | 0.688 | 0.658 | 2.173 | 0.141 | 1.15 (0.96–1.38) | 0.134 |

SNP, single nucleotide polymorphism; OR, odds ratio; 95% CI, 95% confidence interval.

*p^a^* values were calculated byχ^2^.

*p^b^* values were calculated by logistic regression analysis with adjustments for age and gender.
